# Case of Complete Remission from Palliative Radiation in Extensive Stage Small Cell Lung Cancer With Large Brain Metastasis Nine Years After Diagnosis: Cure Is Possible

**DOI:** 10.7759/cureus.10011

**Published:** 2020-08-25

**Authors:** Ming Pan

**Affiliations:** 1 Radiation Oncology, Windsor Regional Hospital Cancer Program, Windsor, CAN

**Keywords:** extensive stage small cell lung cancer, brain metastasis, whole brain irradiation, neurotoxicity, multimodality treatment, consolidative thoracic radiotherapy, complete remission

## Abstract

Extensive stage small cell lung cancer (ES-SCLC) with symptomatic brain metastasis (BM) carries very poor prognosis with a median survival of only three to four months. This case shows the potential benefit of offering aggressive multimodality treatment to achieve the best quality of life and to improve survival. A complete response (CR) was achieved following craniotomy, whole brain irradiation (WBI), palliative chemotherapy, and palliative consolidative thoracic radiotherapy (CTRT) for ES-SCLC with large symptomatic BM. There was no residual cancer or further metastasis seen on CT imaging. The patient remains cancer free and asymptomatic nine years after the initial diagnosis.

## Introduction

Lung cancer is the leading cause of cancer death in Canada, accounting for 25.5% of all cancer deaths [1]. Small cell lung cancer (SCLC) is the second most common thoracic malignancy, representing approximately 13% of newly diagnosed lung cancers [2]. Extensive stage small cell lung cancer (ES-SCLC) with symptomatic brain metastasis (BM) carries very poor prognosis with a median survival of only three to four months from the time of diagnosis [3]. They are usually treated primarily with palliative whole brain irradiation (WBI) followed by systemic therapy [4]. The goals of treatment are to minimize toxicity and to maximize both survival and quality of life (QoL) [5].

Craniotomy is rarely performed for symptomatic BM followed by palliative WBI, which has not been proven to improve survival. WBI therapy with or without stereotactic radiosurgery (SRS) boost for patients with a limited number of BM has been used in phase III clinical trials, as well as SRS with or without WBI in a similar population. Unfortunately, they have limited data in ES-SCLC overall survival (OS) [6-10].

We report a complete response (CR) following craniotomy, WBI, palliative chemotherapy, and palliative dose radiation to the primary cancer in lung and mediastinum for ES-SCLC with large symptomatic BM. There was no residual cancer seen on follow-up CT imaging. The patient remains cancer free and asymptomatic with no significant neurotoxicity or lung toxicity nine years after the initial diagnosis.

## Case presentation

**Figure 1:**
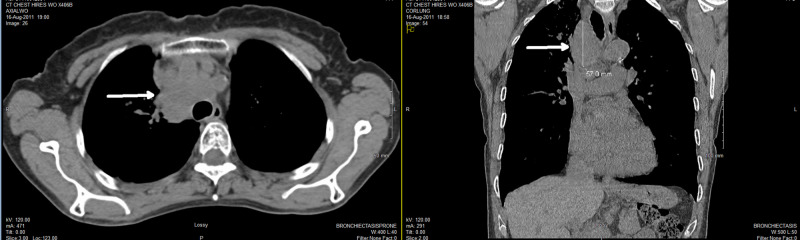
Initial diagnostic CT of the chest showing large masses in the right upper lobe lung and mediastinum

A 71-year-old ex-smoker female presented with respiratory symptoms, including cough and shortness of breath. Other comorbidities included chronic obstructive pulmonary disease (COPD), emphysema, chronic renal failure, and history of ovarian cancer cured by surgery in 1982. CT of the chest showed large masses in the right upper lobe lung and mediastinum (Figure 1). The largest right paratracheal lymph node measured 3.6 x 4.0 x 5.7 cm in size with necrotic center. Bronchoscopy and transbronchial needle biopsy from subcarinal lymph node were done, but pathology report was not immediately available.

**Figure 2:**
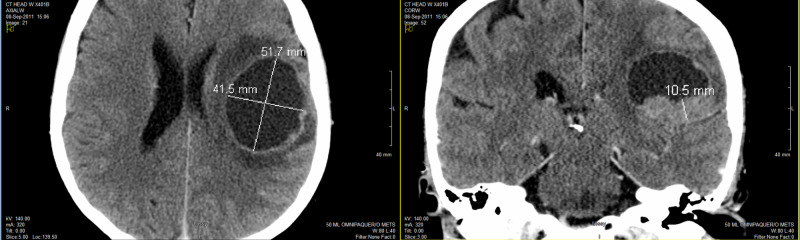
CT of the head showing a large 5.2-cm mass in left parietal brain causing extensive vasogenic edema and midline shift to the right side

Due to her severe headaches, expressive aphasia, and right-sided weakness, a CT of the head was ordered and showed a large 5.2-cm mass in left parietal brain, causing extensive vasogenic edema and midline shift to the right side (Figure 2). She had craniotomy done for these neurological symptoms on September 11, 2011. Frozen-section pathology showed metastatic poorly differentiated carcinoma. However, the final pathology confirmed metastatic small cell carcinoma consistent with lung primary. Immunohistochemistry staining showed the cancer cells positive for synaptophysin, CD56, and thyroid transcription factor-1 (TTF-1). They were negative for pan-cytokeratin and glial fibrillary acidic protein (GFAP). Further CT staging workup did not find any other distant metastasis outside of the brain.

**Figure 3:**
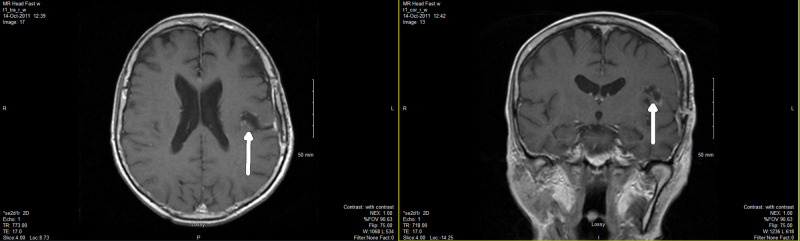
Post-WBI MRI showing no residual disease in the surgical bed or other part of the brain WBI, whole brain irradiation

She received a palliative course of radiation to the whole brain, 20 Gy in five fractions with the last dose on October 7, 2011 (Figure 3). She tolerated the treatment well with no significant side effects. Then she completed four cycles of chemotherapy in the form of cisplatin and etoposide (CE) with the last dose in January 2012. Initially, she had a very good partial response (PR) with the primary cancer shrinking in size. CT scan from February 3, 2012 showed a small residual paratracheal lymph node measuring 2.3 x 2.46 cm in size. Clinically, she was doing well with no symptoms and gained some weight back. Her ECOG (Eastern Cooperative Oncology Group) score remained at 1. The decision was to defer palliative radiation to any recurrent primary lung mass and mediastinal lymph nodes for 20 Gy in five fractions, but only if she had more respiratory symptoms.

**Figure 4:**
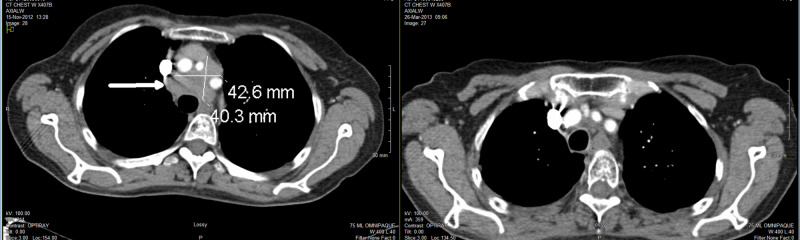
CT in November 2012 showing recurrent mass in mediastinum (image on left) and in March 2013 showing CR after palliative CTRT 30 Gy (image on right) CR, complete response; CTRT, consolidative thoracic radiotherapy

Her cancer recurred and progressed to a large mass 4.9 x 4.2 x 4.0 cm in the right suprahilar, paratracheal, and precarinal regions on CT scan in November 2012 (Figure 4, left image). She had some minimal respiratory symptoms including cough and shortness of breath. We offered her slightly higher dose palliative radiation to the primary cancer and right hilar/mediastinal lymph nodes with 30 Gy in 10 fractions over two weeks. No higher dose CTRT was used in the fear of lung toxicity. She completed the last dose on January 30, 2013. Follow-up CT showed CR with no evidence of any metastasis (Figure 4, right image).

**Figure 5:**
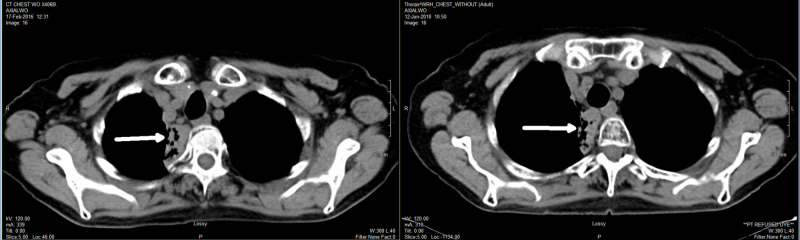
Follow-up CT showing CR with stable post-radiation changes in right lung in 2016 (left) and 2018 (right) CR, complete response

**Figure 6:**
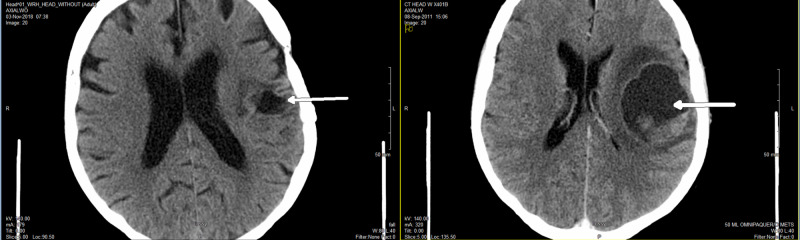
CT scan in November 2018 showing CR in brain (left) comparing to the original CT in 2011 (right) CR, complete response

**Figure 7:**
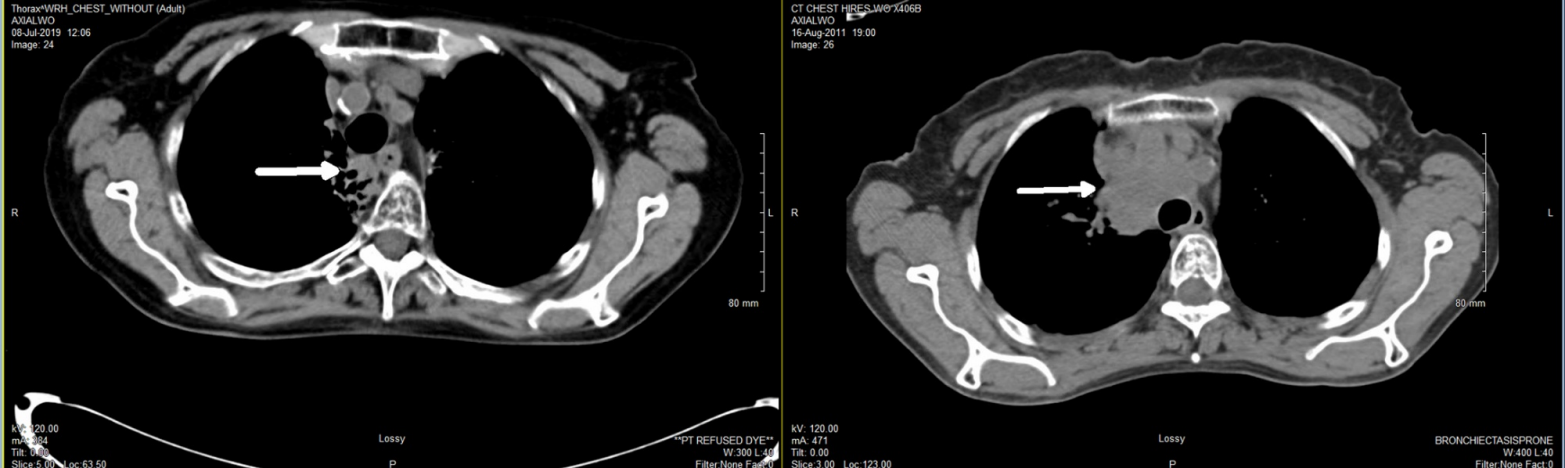
CT in July 2019 showing no signs of recurrence with stable post-radiation changes in the right lung (left) comparing to the original CT in 2011 (right)

She refused to be discharged from the cancer clinic after a five-year cancer-free period, but accepted annual CT scan and follow-up (Figures 5, 6). Unfortunately, she fell and fractured her right hip in the winter of 2019, but she quickly recovered after an open surgery with internal fixation despite her age. Her last CT in July 2019 did not show any signs of recurrent lung cancer or metastasis (Figure 7). There were stable post-radiation changes with some fibrosis in the right upper lobe lung.

Nine years after the diagnosis, the patient was still alive with good performance status and no signs of relapse. Her ECOG was 1 due to some shortness of breath from her baseline COPD. She denied any cough, chest pain, or hemoptysis. She had no neurotoxicity. At the age of 80 years, she lived alone independently, maintained all daily activities, and also helped out her extended family. She was able to drive her grandchildren to all kinds of activities until the lockdown for coronavirus disease 2019 (COVID-19) pandemic. She remained healthy and did not have any signs of COVID-19 infection on the last follow-up visit in July 2020. Her most recent scheduled CT scan had to be cancelled due to hospital pandemic policy but she received virtual care through Ontario Telemedicine Network. Again, she insisted to be seen on yearly basis.

## Discussion

Lung cancer is a deadly disease. It is estimated that 29,800 Canadians will be diagnosed with lung cancer in 2020 and that 21,200 men and women will die from the disease in the same year [1]. SCLC represents about 13% of newly diagnosed lung cancers [2]. Approximately 10% to 15% of patients present with BM and approximately 60% of patients with SCLC develop BM. It happens so often that prophylactic cranial irradiation (PCI) has shown survival benefit in both clinical trials for patients with limited stage SCLC (LS-SCLC) in complete remission after combined modality treatment (CMT) and ES-SCLC after chemotherapy [11,12].

ES-SCLC with BM carries very poor prognosis with a median survival of only three to four months from the time of diagnosis. WBI gives symptomatic improvement in more than 50% of these patients [3]. They are usually treated primarily with cisplatin-based palliative chemotherapy following WBI [4]. The goal is to achieve better local control and QoL [5]. Unfortunately, there is no significant improvement in OS after CMT in the past, in contrast to LS-SCLC [13]. Radiation oncologists and patients are reluctant to consider high-dose consolidative thoracic radiotherapy (CTRT) due to the concern of severe side effects and worse QoL. Koshy et al. concluded that patients with private insurance and treated in community cancer centers seemed more likely to receive longer courses of radiation or CMT compared with academic programs. They considered these patients requiring palliative thoracic radiotherapy were overtreated [14]. National Comprehensive Cancer Network (NCCN) Clinical Practice Guidelines suggested CTRT is only reserved for ES-SCLC without BM and with low-bulk extrathoracic metastatic disease that has responded to systemic therapy. Only palliative WBI was recommended, and there was no recommendation regarding craniotomy [4].

There are rare case reports of long-time survivors after CMT including radical dose radiation to both the brain and thorax, but no cases have been reported for ES-SCLC cured with only palliative doses of radiation to the brain and lungs, with no significant neurotoxicity or lung toxicity achieving a nine-year survival [15,16]. Some researchers believe that in ES-SCLC BM was not a negative prognostic factor if the patients were treated appropriately [17]. Lekic et al. reported the outcome of 251 cases of SCLC with BM. All patients received chemotherapy and all patients with confirmed BM (either at initial diagnosis or during follow-up) received WBI. The prognosis of their patients with ES-SCLC with BM at the primary diagnosis treated with chemotherapy and WBI was not significantly worse than that of patients with ES-SCLC and metastases outside the brain. Median OS was 9 months for 34 patients with BM, 11 months for 153 patients with metastases in other locations (p = 0.62), and 15 months for 64 patients without metastases at the time of primary diagnosis (p < 0.001). However, only 26 (10.4%) patients received CTRT and only 17 (6.8%) patients underwent PCI which had not become standard at the time of the study but should have increased the difference of OS in the first two subgroups of their cohort [17]. More aggressive treatment should have been offered based on current guidelines [4,18].

Jeremic et al. studied the role of CMT in patients with ES-SCLC in a randomized trial. A total of 210 patients were treated with three cycles of standard CE chemotherapy. Patients with CR at both the local and distant levels or a PR at the local level and CR at the distant level received either CTRT with 54 Gy in 36 fractions over 18 treatment days in combination with CE followed by two more cycles chemotherapy (n = 55) or an additional four cycles of chemotherapy (n = 54). All patients with a CR at the distant level received PCI. CTRT achieved significantly better survival rates than those with chemotherapy alone (median OS, 17 vs 11 months; five-year OS, 9.1% vs 3.7%, respectively; p = 0.041). Acute high-grade toxicity was higher in chemotherapy group than in radiation group [19]. Their conclusion was that the addition of CTRT to the treatment of the most favorable subset of ES-SCLC patients led to improved survival over that obtained with chemotherapy alone. This was further confirmed by the American Society for Radiation Oncology (ASTRO) guidelines, but the recommended dose was 30 Gy in 10 fractions [18].

Most patients who received WBI reported severe long-term neurotoxicity, so SRS and hypofractionated stereotactic radiotherapy (HSRT) have become popular in recent years, with multiple clinical trials and meta-analysis showing some improvement in survival and QoL [6-10]. But these studies do not have a subgroup analysis to show any benefit of SRS in ES-SCLC. If PCI has been widely accepted due to survival benefit from controlling microscopic BM, then SRS alone should not be encouraged in ES-SCLC at all. Chang et al. reported in a randomized controlled trial that 73% of patients in the SRS plus WBI group were free from CNS recurrence at one year, compared with 27% of patients who received SRS alone (p = 0.0003). However, their trial was stopped early when interim data analysis showed that there was a high probability (96%) that patients receiving SRS plus WBI were significantly more likely to show a decline in learning and memory function at four months than patients assigned to receive SRS alone (mean posterior probability of decline 52% vs 24%) [10].

Our institution started HSRT for BM in 2018, with current protocol to offer up to 30-Gy HSRT in five fractions every other day. However, we have been always offering WBI alone to SCLC patients, either 25-Gy PCI in ten daily fractions or 20-Gy WBI in five daily fractions. If patients survive more than 12 months after the first course PCI or WBI, we do offer a second course of 21-Gy WBI in seven fractions for symptomatic progression of BM [20]. We also offer HSRT boost to selected patients with persistent or recurrent solitary BM after WBI. This patient was a surprise to us with excellent response to only one course of 20-Gy WBI after craniotomy. She had no extrathoracic metastasis other than the brain. When her primary cancer recurred after four cycles of palliative CE chemotherapy, we decided to offer her CTRT. She was aware of the possible severe lung toxicities, which never happened in her case, and consented to lower dose 30 Gy in 10 fractions. Fortunately, this achieved excellent local control and CR. We also kept her QoL.

It should be noted that we did not offer her CMT at the initial diagnosis of ES-SCLC. She was unlikely to have tolerated the toxicities of high-dose concurrent chemoradiation due to her age and comorbidities, including COPD, emphysema, and chronic renal failure. But for younger patients with good performance status, some previous studies have shown good results [19]. Therefore, not every patient should undergo standard palliative approach, as recommended in the published guidelines [4,18]. However, this case might also be considered as LS-SCLC with oligometastasis in the brain. It is different from other ES-SCLC with multiple metastases in several organs, so MMT can give a long-term control of the disease.

To our knowledge, this is the first report of ES-SCLC with large symptomatic BM cured with just palliative dose radiation to both the brain and primary cancer in the thorax.

## Conclusions

ES-SCLC with solitary BM can have better prognosis than those with widespread extracranial distant metastases. Long-term local control and survival is rare but still possible even after palliative dose radiation treatment. Not all patients will have severe neurotoxicity and lung toxicity after WBI and CTRT. Good QoL is achievable. Aggressive MMT should be considered for highly select patients with good performance status. Some of the terminal stage SCLC might be cured.
